# Supplementary material for: Negative emotional reactions to criticism: Perceived criticism and source affects extent of hurt and relational distancing
Source: PLoS One. 2022 Aug 8;17(8):e0271869. doi: 10.1371/journal.pone.0271869 (PMC9359543; doi:10.1371/journal.pone.0271869)
Supplement: Attachment — Submitted filename: HurtCrit_Review2_response.docx [file pone.0271869.s002.docx]

Reviewer #1: My initial review was extensive, and the authors have done a commendable job of addressing my concerns. However, two significant concerns remain:

Thank you for your comments. Please find our responses to your comments below:

1. In response to my comments about assuming closeness based on type of relationship, the authors noted that they controlled for relationship quality. However, relationship quality is not the same as closeness. I could have a really fantastic (high quality) relationship with my mailman but not be very close to him. Similarly, I could have a really terrible relationship (low quality) with my sister, but also be very close to her (e.g., situations of abuse, co-dependency, enmeshment). Controlling for relationship quality does not address the issues of assuming closeness based on relationship type. Relatedly, the authors do not include any information about how they measured relationship quality, which is a significant omission.

We have included information on how relationship quality was measured as follows: “Relationship quality was measured using questions adapted from the Quality of Marriage Index [40]. Participants rated the quality of each of the relationship type: romantic partner, mother, father and workplace supervisor. There were 5 items rated on a 7-point Likert scale and 1 item rated on a 10-point Likert scale where higher scores reflected higher relationship quality.”

We agree that relationship quality and relationship closeness are different and we acknowledge the assumption of closeness based on relationship type in the limitations as follows: “The analysis of the present study was also assumed relationship closeness based on the relationship type where familial and intimate relationships were assumed to be close compared to the relationship with a supervisor. Future studies can include measures of relationship closeness to examine whether experiences of relationship closeness also play a role in the response towards criticism originating in different relationship types.”

2. In response to my comments about using a median split, the authors changed their analytic approach. I appreciate this effort (although I could have been easily convinced that a median split was appropriate given the precedent in the PC literature—I was mostly suggesting greater justification). However, the analysis is now confusing and seems disjointed. They still use F statistics and refer to PC groups (e.g., Tables 2a and 2b and the associated text). Table 4 also still refers to low and high PC groups.

Thank you for your comment. We have used the median split for PC and updated the manuscript accordingly.

We have also cited the use of the median split in previous PC literature as follows: “Participants were grouped into high or low on PC through a median split (MdnMother = 4, MdnFather, MdnPartner, MdnSupervisor = 5). The medians in this sample are also similar to previous studies on PC which have also used median splits (e.g. [12] and [41]) to facilitate interpretation of results.”
